# Diagnostic performance of preoperative [^18^F]FDG-PET/CT for lymph node staging in vulvar cancer: a large single-centre study

**DOI:** 10.1007/s00259-021-05257-8

**Published:** 2021-02-23

**Authors:** Vittoria Rufini, Giorgia Garganese, Francesco P. Ieria, Tina Pasciuto, Simona M. Fragomeni, Benedetta Gui, Anita Florit, Frediano Inzani, Gian Franco Zannoni, Giovanni Scambia, Alessandro Giordano, Angela Collarino

**Affiliations:** 1 Section of Nuclear Medicine, University Department of Radiological Sciences and Haematology, Università Cattolica del Sacro Cuore, Largo A. Gemelli, 8, 00168 Rome, Italy; 2 Unit of Nuclear Medicine, Fondazione Policlinico Universitario A. Gemelli IRCCS, Rome, Italy; 3 Department of Woman and Child Health and Public Health, Vul.Can MDT, Fondazione Policlinico Universitario A. Gemelli IRCCS, Rome, Italy; 4 Gynecology and Breast Care Center, Mater Olbia Hospital, Olbia, Italy; 5 Unit of Radiology, Fondazione Policlinico Universitario A. Gemelli IRCCS, Rome, Italy; 6 Unit of Gynecopathology, Department of Woman and Child Health and Public Health, Fondazione Policlinico Universitario A. Gemelli IRCCS, Rome, Italy; 7 Section of Pathology, Department of Woman and Child Health and Public Health, Università Cattolica del Sacro Cuore, Rome, Italy; 8 Section of Obstetrics and Gynecology, University Department of Life Sciences and Public Health, Università Cattolica del Sacro Cuore, Rome, Italy

**Keywords:** [^18^F]FDG-PET/CT, Vulvar cancer, Lymph node staging, Personalized medicine

## Abstract

**Purpose:**

This retrospective study aimed to assess the diagnostic performance of preoperative [^18^F]FDG-PET/CT in predicting the groin and pelvic lymph node (LN) status in a large single-centre series of vulvar cancer patients.

**Methods:**

Between January 2013 and October 2018, among all consecutive women with proven vulvar cancer submitted to [^18^F]FDG-PET/CT, 160 patients were included. LNs were analysed by two qualitative methods assessing PET information (defined as *visual assessment*) and a combination of PET and low-dose CT information (defined as *overall assessment*), respectively, as well as semi-quantitative analysis (LN-SUV_max_). Sensitivity, specificity, accuracy, positive and negative predictive values (PPV and NPV) in predicting the groin and pelvic LN status were calculated in the overall study population; a subset analysis of groin parameters in clinically/ultrasonography negative patients was also performed. Histopathology was the reference standard.

**Results:**

All patients underwent vulvar and inguinofemoral LN surgery, and 35 pelvic LN surgery. Overall, 338 LN sites (296 groins and 42 pelvic sites) were histologically examined with 30.4% prevalence of metastatic groins and 28.6% for metastatic pelvic sites. In the overall study population, sensitivity (95% confidence interval, CI), specificity (95% CI), accuracy (95% CI), PPV (95% CI) and NPV (95% CI) at the groin level were 85.6% (78.3–92.8), 65.5% (59.0–72.0), 71.6% (66.5–76.8), 52.0% (44.0–60.1) and 91.2% (86.7–95.8) for *visual assessment*; 78.9% (70.5–87.3), 78.2% (72.5–83.8), 78.4% (73.7–83.1), 61.2% (52.3–70.1) and 89.4% (85.0–93.9) for *overall assessment*; and 73.3% (64.2–82.5), 85.0% (80.1–89.8), 81.4% (77.0–85.8), 68.0% (58.8–77.3) and 87.9% (83.4–92.5) for semi-quantitative analysis (SUV_max_ cut-off value 1.89 achieved by ROC analysis). Similar results were observed in the pelvis-based analysis.

**Conclusion:**

In this large single-centre series of vulvar cancer patients, [^18^F]FDG-PET/CT showed good values of sensitivity and NPV in discriminating metastatic from non-metastatic LNs. In routine clinical practice, qualitative analysis is a reliable interpretative criterion making unnecessary commonly used semi-quantitative methods such as SUV_max_.

**Supplementary Information:**

The online version contains supplementary material available at 10.1007/s00259-021-05257-8.

## Introduction

Vulvar cancer is a rare gynaecological tumour with an estimated incidence of 2.6 new cases/100,000 women per year [1]. The most common histological type is squamous cell carcinoma [2]. The major pathway of spread is to the inguinofemoral and then to the pelvic lymph nodes (LNs), whereas haematogenous spread is rare [3]. Surgery is the most common treatment, varying from minimally invasive surgery to extensive excision, often requiring plastic reconstruction [4–6]. LN involvement is the main prognostic factor [7]. Therefore, an accurate LN assessment is crucial to personalize the surgical plan. Surgery varies from the minimally invasive sentinel node biopsy (SNB) to the more demolitive radical lymphadenectomy, which is often followed by lymphedema of the lower limbs [8]. LN assessment is mainly performed by ultrasonography with or without cytological evaluation of suspicious LNs and computed tomography (CT) [9–12]. Positron emission tomography/CT with [^18^F]fluorodeoxyglucose ([^18^F]FDG-PET/CT) has been included in the National Comprehensive Cancer Network guidelines for vulvar cancer since 2016 and is recommended for T2 or larger tumours or when metastases are suspected [4]. However, only few studies on small series with controversial results on the role of [^18^F]FDG-PET or PET/CT in vulvar cancer have been published [12–21]; they have been summarized in a recent meta-analysis [22].

This study aims to assess the diagnostic performance of preoperative [^18^F]FDG-PET/CT in predicting the groin and pelvic LN status in a large single-centre series of vulvar cancer patients.

## Materials and methods

### Patients and study design

This was a single institution, retrospective study that was approved by the local Ethics Committee (ID 3000). All patients signed a written informed consent, and their data were collected using Research Electronic Data Capture (REDCap) tool.

Between January 2013 and October 2018, all consecutive women with histologically proven invasive (depth of stromal invasion > 1 mm) vulvar cancer with any stage and any histology, who underwent preoperative [^18^F]FDG-PET/CT at our institution, were included. Exclusion criteria were prior inguinofemoral dissection, previous chemotherapy or radiotherapy, distant metastases outside the pelvis at staging [^18^F]FDG-PET/CT, contraindications to surgery due to age or comorbidities, surgery performed > 40 days after [^18^F]FDG-PET/CT and recurrent tumour.

Patients were staged according to FIGO Stage 2009 [23] and were evaluated by clinical and imaging examinations, including CT scan of the thorax, abdomen and pelvis; [^18^F]FDG-PET/CT; inguinofemoral ultrasonography with or without fine needle aspiration cytology/biopsy of the suspicious LNs on the basis of imaging patterns; MRI, in selected cases. After completing the staging work-up, clinical and imaging data were discussed during the multidisciplinary team (Vul.Can MDT), reaching a consensus on staging and therapeutic approach, according to international guidelines. Histopathology was the reference standard to assess LN metastases.

### [^18^F]FDG-PET/CT

PET/CT scan was performed as previously reported [17]. Patients fasted for 6 h, had glucose blood levels < 200 mg/dl before [^18^F]FDG injection and were hydrated with 500 ml of saline solution. PET/CT images were acquired on a hybrid scanner (Gemini GXL, Philips Medical System, or Biograph mCT Siemens Medical Solutions) at 60 ± 10 min after [^18^F]FDG injection (120–330 MBq according to body weight). Low-dose CT scan (120 keV, 40–50 mAs) was acquired from skull base to the mid thighs for anatomical localization and attenuation correction. All PET images were acquired (2.5–3 min/bed position) in the range defined by CT. For the Siemens Biograph mCT, 3D OSEM reconstruction with PSF modelling/TOF (2 iterations and 21 subsets, voxel size of 3.2 × 3.2 × 5 mm^3^) was applied; the kernel of the Gaussian filter was 2.0 mm. For the Philips Gemini GXL, LOR RAMLA reconstruction (2 iterations and 24 subsets, voxel size: 4 × 4 × 4 mm^3^) was applied; the kernel of the Gaussian filter was 5.0 mm [24].

### Image analysis

PET/CT scans were reviewed by two nuclear medicine physicians (FI and AC, with more than 2 and 5 years of clinical experience in PET/CT imaging, respectively) blinded to clinical and histopathologic information, who reached a consensus. In case of disagreement, the consensus was defined by the senior investigator (VR, with more than 10 years of clinical experience in PET/CT). [^18^F]FDG uptake of the inguinofemoral and pelvic LNs was analysed by qualitative and semi-quantitative methods.

#### Qualitative analysis

The two-side groin and pelvic regions were analysed for each patient. When multiple LNs showing [^18^F]FDG uptake were evident in a single site, the LN with higher activity was considered (index node). Firstly, a score was assigned considering the LN uptake with respect to the gluteus muscle taken as background [17], and liver activity. In particular: *score 0* = no uptake; *score 1* = uptake >background and ≤liver activity; *score 2* = uptake >liver activity. A LN site with score 0 was interpreted as normal, one with score 1 or 2 as abnormal. This interpretative criterion was called *visual assessment*. Subsequently, both [^18^F]FDG uptake and CT appearance of the LNs including size, shape and density were considered. The size of the largest LN was taken on the transaxial CT images (short axis in mm) of PET/CT. The overall decision was taken according to the criteria that are reported in Table 1. Clearly, normal and inflammatory LNs were classified as “non-metastatic with high probability”, and suspicious and clearly abnormal LNs were classified as “metastatic with high probability”. This interpretative criterion was called *overall assessment*.

**Table 1:** Lymph node interpretation according to *overall assessment*

Judgment	^18^F-FDG uptake scoring	Short axis at CT	Shape	Fatty hilum
Clearly normal	0	< 10 mm	Elliptical	Present*
Inflammatory	1	Any size	Elliptical	Present
Suspicious	1–2	< 8 mm	Round	Absent
Clearly abnormal	2	≥ 8 mm	Round	Absent

*Uncertain identification for lymph nodes < 5 mm in short axis diameter

#### Semi-quantitative analysis

A spherical volume of interest was placed over the LN with the highest uptake both for groin and pelvic regions. LN-SUV_max_, defined as the maximum activity within the LN normalized to the injected dose and patient’s body weight, was measured only in case of abnormal LNs at visual analysis, by applying the EQ∙PET reference-based quantification technology in order to harmonize SUV values obtained by two different PET systems [25]. An 8-mm Gaussian filter was applied for the Biograph mCT system, while no EQ∙PET Gaussian filter was needed for GXL system that is not equipped with PSF modelling. SUV_max_ of LNs with normal pattern at visual analysis was set at background SUV_max_ (on the gluteus muscle) both for groin and pelvic sites, for each patient. [^18^F]FDG-PET/CT qualitative and semi-quantitative findings were compared to histopathology for the inguinofemoral and pelvic LNs, separately.

### Surgical procedure

All patients underwent vulvar surgery, which consisted of radical resection of the primary lesion by partial (lateral, anterior or posterior) or total vulvectomy (simple or radical), with macroscopic resection margins > 2 cm. SNB or inguinofemoral lymphadenectomy were chosen based on clinical assessment and standard criteria [4, 26]. They were performed mono- or bilaterally according to the distance of the primary tumour from midline (> or < 2 cm, respectively). In locally advanced disease when chemo-radiation could not be administered or in case of suspicious LNs requiring histologic confirmation, pelvic lymphadenectomy was performed. Histopathology was made by a skilled gynaecologic-oncology pathologist.

### Statistical analysis

Sample size was calculated according to Hajian-Tilaki [27]. Assuming 85.0% sensitivity and 80.0% specificity for [^18^F]FDG-PET/CT in predicting positive groin LNs at histology, as well as a pathological groin LN prevalence of 35%, a precision of estimate (i.e. the maximum marginal error) *d* = 7% and a type I error *α* = 0.05, a sample size of 286 and 193 groins was needed to test our hypothesis according to sensitivity and specificity, respectively. The greatest number of 286 groins was considered as the study protocol sample size. Both groin and pelvic sites were divided in two groups: those with positive and those with negative LNs at histopathology. Results are presented as absolute frequency (percentage) for nominal variables, as mean ± SD (Standard Deviation) for normally distributed continuous variables or normally distributed after transformation and as median (min–max) for continuous variables not normally distributed. Shapiro-Wilk test was used to assess the normality of variable distribution. Comparisons between histopathological groups were made with *t* Student or Mann-Whitney test for continuous variables and with *χ*^2^ or Fisher’s exact test for nominal variables as appropriate. Two-sided tests were used, and the significance level was set at *p* < 0.05. Sensitivity, specificity, accuracy, positive and negative predictive values (PPV and NPV) of [^18^F]FDG-PET/CT in predicting groin and pelvic LN status were calculated. All the parameters were presented with two-sided 95% confidence intervals (CIs). Diagnostic performances of PET parameters were evaluated in the overall study population for groin and pelvic sites; a subset analysis of groin parameters in clinically/ultrasonography negative patients (cN0) was also performed. Receiver operating characteristic (ROC) curves were generated for SUV_max_ to evaluate its ability to predict pathological positive groin or pelvic LNs in terms of area under the curve (AUC) and 95% CI, as well as to determine the best cut-off value to predict positive groin or pelvic LNs versus negative ones at histopathology. Best cut-off was detected according to Youden method [28]. *Z* test was used to compare the performances of *visual assessment*, *overall assessment* and SUV parameters in predicting positive LNs at histopathology. Bonferroni correction was used when appropriate to correct for multiple testing and the significance level was set at *p* < 0.017. All statistical calculations and plots were performed using the Stata software version 13.0 (Stata Corp, College Station, TX).

## Results

**Fig. 1:**
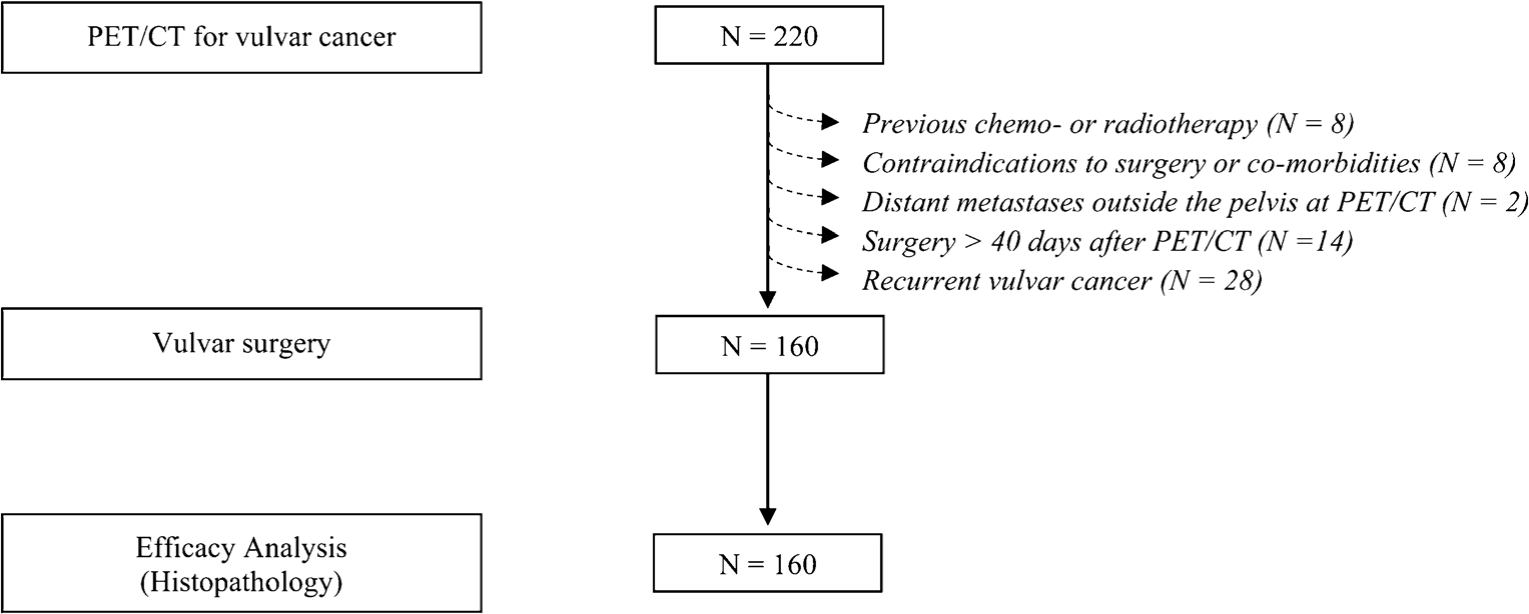
The flowchart of study population

Of 220 patients with proven invasive vulvar cancer submitted to [^18^F]FDG-PET/CT between January 2013 and October 2018, 160 patients fulfilled the inclusion criteria (Fig. 1). Clinical, surgical and pathologic features of patients included are reported in Table 2. No patient with stage IV disease had distant metastases outside the pelvis, according to inclusion/exclusion criteria. The median time interval between preoperative [^18^F]FDG-PET/CT and surgery was 22 days (range 4–40). All patients underwent vulvar surgery and inguinofemoral LN surgery. Thirty-five women underwent additional pelvic LN surgery. Overall, 338 LN sites underwent surgery (296 groins and 42 pelvic sites) and were histologically examined. In the overall study population, the prevalence of metastatic groins was 30.4% (90/296) and that of metastatic pelvic sites 28.6% (12/42). In the subgroups of cN0 cases (96 patients), the prevalence of metastatic groins was 13.9% (24/173). A total of 2495 LNs were removed and analysed (2270 in the groins and 225 in the pelvis). The number of positive LNs at histopathology was 195 in the groins and 44 in the pelvis.

**Table 2:** Clinical and histological characteristics of patients included

Characteristic	All patients (*n* = 160)
Age, year	70.6 ± 12.6 (16–96)
Clinical presentation	
cN0	96 (60.0)
cN+	64 (40.0)
Histotype	
Squamous	143 (89.4)
Other	17 (10.6)
FIGO stage 2009 [23]	
I	86 (53.8)
II	4 (2.5)
III (a–c)	57 (35.6)
IV	13 (8.1)
IVA	2 (1.2)
IVB	11 (6.8)
Focality*	
Unifocal	142/157 (90.4)
Multifocal	15/157 (9.6)
Size of primary lesion**	
< 40 mm	102/155 (65.8)
≥ 40 mm	53/155 (34.2)
Grading^†^	
G1	12/154 (7.8)
G2	105/154 (68.2)
G3	27/154 (17.5)
Not applicable	10/154 (6.5)
Vulvar surgery	
Partial	44 (27.5)
Simple	15 (9.4)
Radical	89 (55.6)
Ultraradical	12 (7.5)
Inguinal LN surgery	
SNB	33 (20.6)
IFL	120 (75)
SNB + IFL	7 (4.4)
Unilateral	24 (15.0)
Bilateral	136 (85.0)
Pelvic LN surgery	
SNB	16 (45.7)
PL	19 (54.3)
Monolateral	28 (80.0)
Bilateral	7 (20.0)

Results are presented as *n* (%) and mean ± SD (range) for qualitative and quantitative characteristics, respectively. *cN0*, clinically/ultrasonography negative; *cN+*, clinically/ultrasonography positive; *SNB*, sentinel node biopsy; *IFL*, inguinofemoral lymphadenectomy; *PL*, pelvic lymphadenectomy

*Information available for 157 patients

**Information available for 155 patients

^†^Information available for 154 patients

**Fig. 2:**
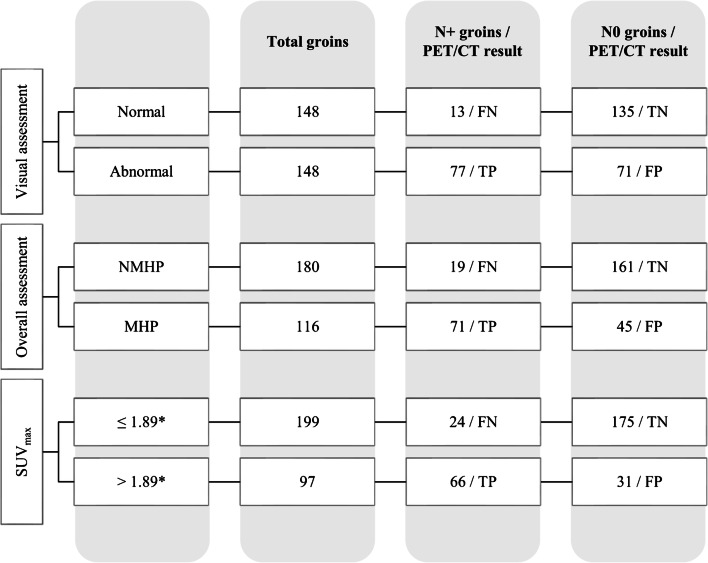
Results of qualitative and semi-quantitative analysis in discriminating positive versus negative LNs at histopathology, for groin sites. FN, false negative; TN, true negative; TP, true positive; FP, false positive; NMHP, non-metastatic with high probability; MHP, metastatic with high probability. *Best cut-off value achieved by ROC analysis

**Fig. 3:**
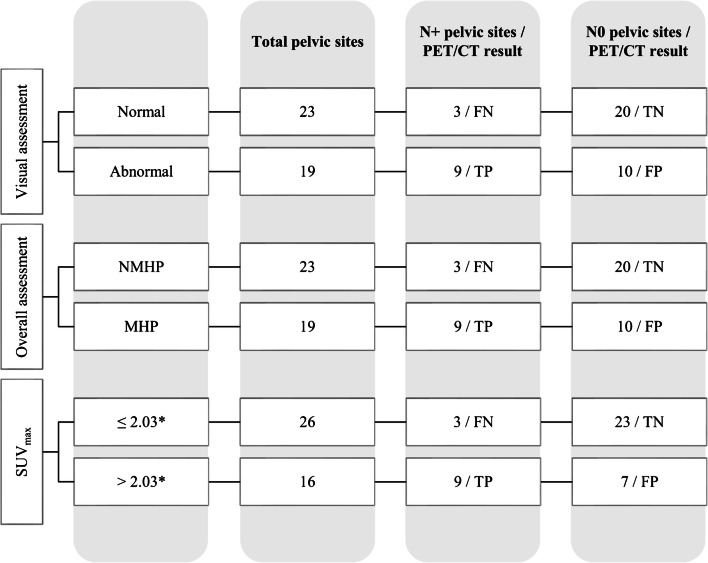
Results of qualitative and semi-quantitative analysis in discriminating positive versus negative LNs at histopathology, for pelvic sites. FN, false negative; TN, true negative; TP, true positive; FP, false positive; NMHP, non-metastatic with high probability; MHP, metastatic with high probability. *Best cut-off value achieved by ROC analysis

Figures 2 and 3 show a synthetic organigram with the results of qualitative (both *visual* and *overall assessment*) and semi-quantitative analysis in discriminating positive versus negative LNs at histopathology in the overall study population for groins and pelvic sites, respectively. In synthesis, in the groin-based analysis, *visual assessment* showed the greatest number of true positive (TP) results (*n* = 77) with respect to *overall assessment* (*n* = 71) and semi-quantitative analysis (*n* = 66). However, it showed the greatest number of false positive (FP) results (*n* = 71) with respect to *overall assessment* (*n* = 45) and semi-quantitative analysis (*n* = 31). In the pelvic-based analysis, both qualitative (*visual* and *overall assessment*) and semi-quantitative analysis showed the same number of true positive (TP) results (*n* = 9). However, qualitative assessment showed the greatest number of FP results (*n* = 10) with respect to semi-quantitative analysis (*n* = 7). Online Resources 1 and 2 show in detail the diagnostic characteristics of groin and pelvic sites, respectively.

**Fig. 4:**
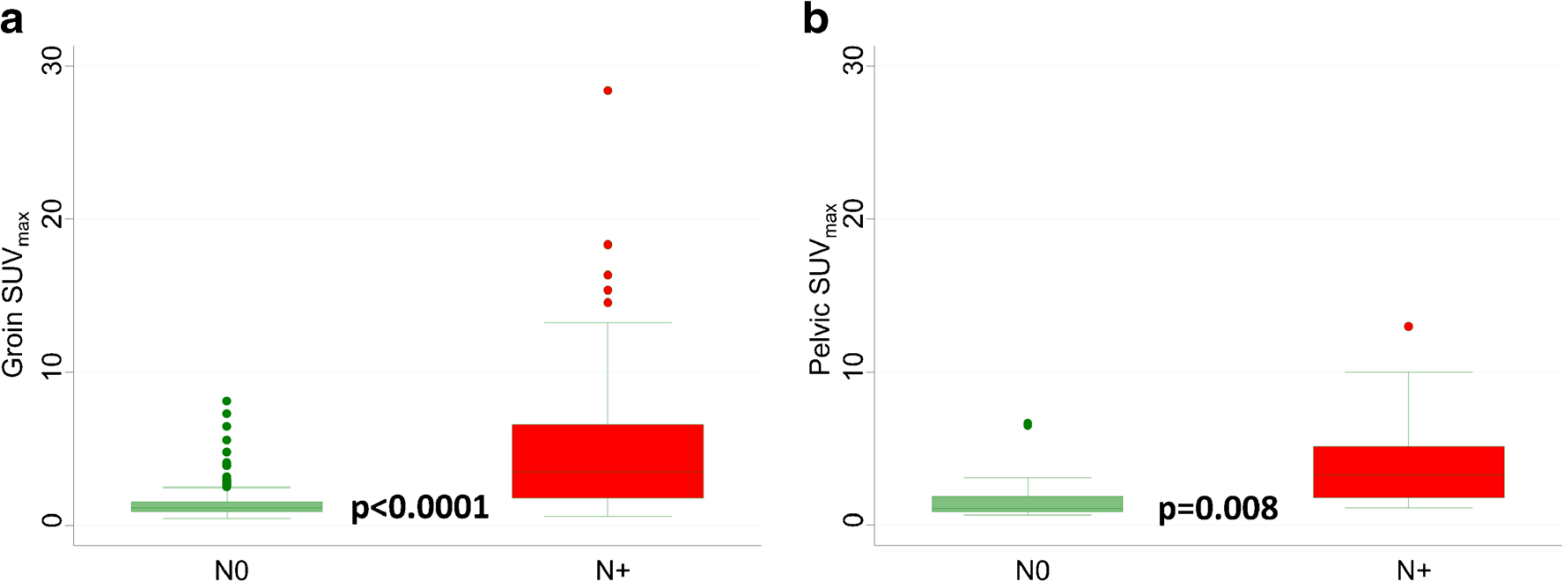
Box plots showing distribution of groin SUV_max_ (**a**) and pelvic SUV_max_ (**b**) for non-metastatic and metastatic LNs. The boxes indicate medians with upper (Q3) and lower quartiles (Q1); the upper and lowers bars define the upper and lower adjacent values, respectively; dots indicate outliers [29, 30]

**Fig. 5:**
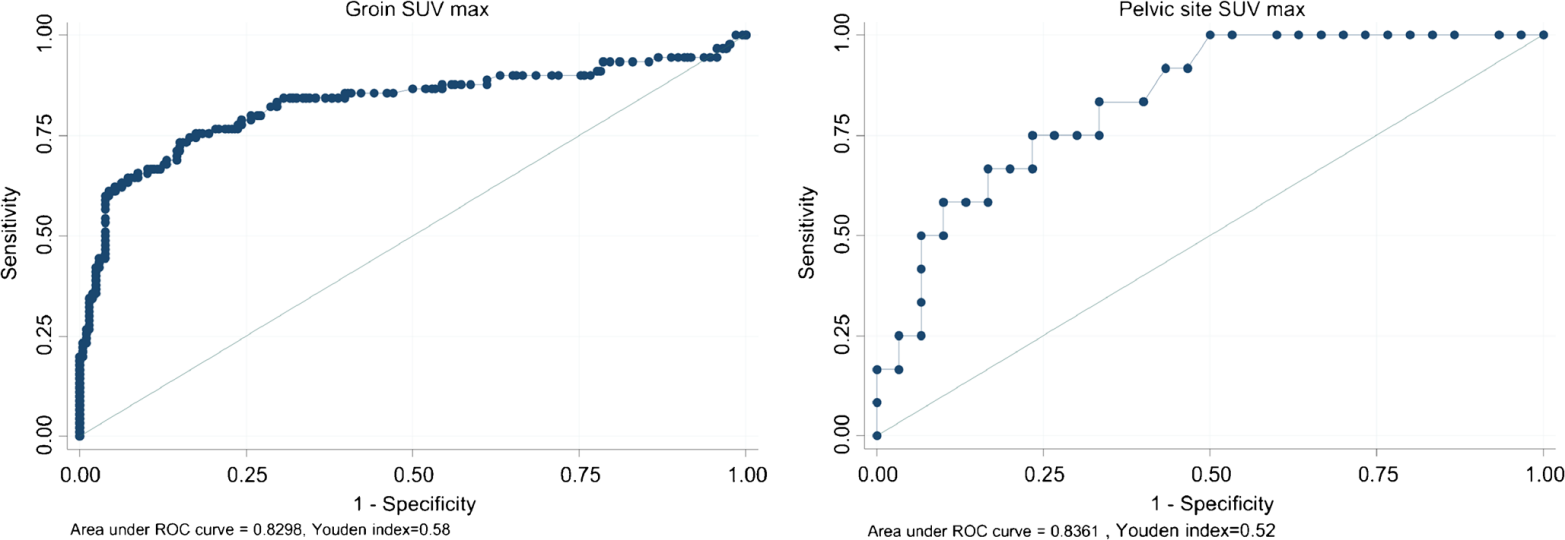
The receiver operating characteristic curves of SUV_max_ for groin and pelvic sites

**Fig. 6:**
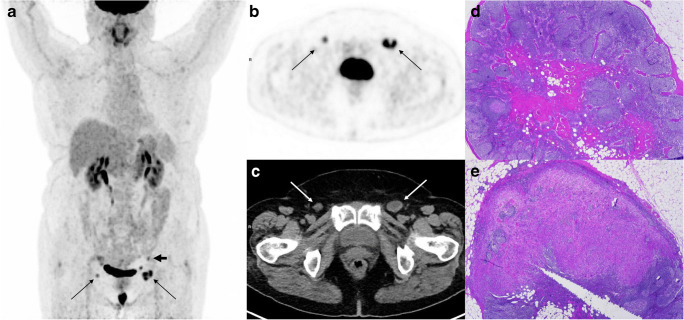
A 68-year-old woman with untreated squamous cell carcinoma. Multiple intensity projection (MIP) images showing ^18^F-FDG uptake in two groin LNs (thin arrows) and one pelvic LN (thick arrow) (**a**). ^18^F-FDG-PET showing focal uptake both in the right (score 2 at *visual assessment*, SUV_max_ 2.5) and in the left (score 2 at *visual assessment*, SUV_max_ 6.0) groin LN (thin arrows) (**b**). At low-dose CT, the right LN shows short axis diameter of 11 mm and round shape, the left LN short axis of 16 mm, round shape and possible necrosis (thin arrows) (**c**). At *overall assessment*, both LNs are judged as clearly abnormal. Pathologic examination showed reactive features in all the right groin LNs removed (**d**) and metastasis in the largest LN among those removed in the left groin (**e**). The pelvic LN, which was located in the obturator region (PET and CT images not shown), was metastatic at histopathology

Table 3 shows the median short axis of positive and negative groin and pelvic LNs at histopathology according to the PET/CT results at *visual assessment* and *overall assessment*. In particular, there was a statistical difference in short axis size between metastatic and non-metastatic nodes both for normal and abnormal LNs at *visual assessment* as well as for clearly normal/inflammatory LNs (non-metastatic with high probability) and suspicious/clearly abnormal LNs (metastatic with high probability) at *overall assessment*. Absolute LN-SUV_max_ values were significantly higher for positive than for negative LNs at histopathology both in the groin-based (Fig. 4a) and in the pelvis-based (Fig. 4b) analysis [29, 30]. In the 90 groins with positive LNs at histopathology, the median SUV_max_ was 3.5 (range 0.6–28.4). In the 12 pelvic sites with positive LNs at histopathology, the median SUV_max_ was 3.7 (range 2.2–13.0); in the same patients, the median SUV_max_ at the groin level was 6.3 (range 2.3–16.3). At ROC analysis, the best cut-off value to predict positive groin or pelvic LNs versus negative ones was 1.89 and 2.03, respectively (Fig. 5). Figure 6 shows [^18^F]FDG-PET/CT images of one patient with 2 groin LNs showing abnormal [^18^F]FDG uptake (score 2), one positive and one negative at histopathology. Table 4 shows the diagnostic performance of the three interpretative criteria both for groin and pelvic sites, obtained in the overall study population and in the subgroup of cN0 patients. The higher values of sensitivity were observed at *visual assessment* in the overall study population (85.6%), markedly dropping in cN0 patients (62.5%). Conversely, similar values of NPV were observed in the overall study population (91.2%) and in cN0 patients (92.2%) at *visual assessment.* The results obtained in the groin- and pelvic-based analysis were compared, and data are shown in Online Resource 3. In synthesis, a significant difference was found between groin LN-SUV_max_ (higher value of specificity) versus *visual assessment* (*p* < 0.00001); between *overall assessment* (higher value of specificity) versus *visual assessment* (*p* = 0.003); and between groin LN-SUV_max_ (higher value of accuracy) versus *visual assessment* (*p* = 0.0101).

**Table 3:** The median short axis of positive and negative groin and pelvic LNs at histopathology according to PET results at *visual assessment* and PET/CT results at *overall assessment*

	Median short axis				
	Negative LNs		Positive LNs		*p* value
	*N*	Median size (min–max)	*N*	Median size (min–max)	
Visual assessment					
Normal					
Groin	135	0 mm (0–12)	13	6 mm (4–8)	**0.0001**
Pelvic site	20	0 mm (0–8)	3	0 mm (0–2)	0.349
Abnormal					
Groin	71	9 mm (5–17)	77	12 mm (5–67)	**0.0001**
Pelvic site	10	6.5 mm (5–11)	9	10 mm (8–25)	**0.003**
Overall assessment					
NMHP					
Groin	161	0 mm (0–14)	19	6 mm (4–10)	**0.001**
Pelvic site	20	0 mm (0–8)	3	0 mm (0–2)	0.349
MHP					
Groin	45	10 mm (5–17)	71	12 mm (5–67)	**0.002**
Pelvic site	10	6.5 mm (5–11)	9	10 mm (8–25)	**0.003**

For *p* value, bold font highlights statistically significant value. *TN*, true negative; *FN*, false negative; *FP*, false positive; *TP*, true positive; *NMHP*, non-metastatic with high probability; *MHP*, metastatic with high probability

**Table 4:** Diagnostic performance of PET/CT parameters in discriminating positive versus negative lymph nodes at histopathology

Characteristics	Area under ROC curve estimate (95% CI)	Cut-off, Youden index*	Sensitivity % (95% CI)	Specificity % (95% CI)	Accuracy % (95% CI)	PPV % (95% CI)	NPV % (95% CI)	TP	TN	FN	FP	Total
All cases												
Groin parameters												
Visual assessment	–	–	85.6 (78.3–92.8)	65.5 (59.0–72.0)	71.6 (66.5–76.8)	52.0 (44.0–60.1)	91.2 (86.7–95.8)	77	135	13	71	296
Overall assessment	–	–	78.9 (70.5–87.3)	78.2 (72.5–83.8)	78.4 (73.7–83.1)	61.2 (52.3–70.1)	89.4 (85.0–93.9)	71	161	19	45	296
LN-SUV_max_	0.83 (0.77–0.89)	1.89, 0.58	73.3 (64.2–82.5)	85.0 (80.1–89.8)	81.4 (77.0–85.8)	68.0 (58.8–77.3)	87.9 (83.4–92.5)	66	175	24	31	296
Pelvic parameters												
Visual assessment	–	–	75.0 (50.5–99.5)	66.7 (49.8–83.5)	69.0 (55.1–83.0)	47.4 (24.9–69.8)	87.0 (73.2–100.0)	9	20	3	10	42
Overall assessment	–	–	75.0 (50.5–99.5)	66.7 (49.8–83.5)	69.0 (55.1–83.0)	47.4 (24.9–69.8)	87.0 (73.2–100.0)	9	20	3	10	42
LN-SUV_max_	0.84 (0.72–0.97)	2.03, 0.52	75.0 (50.5–99.5)	76.7 (61.5–91.8)	76.2 (63.3–89.1)	56.3 (31.9–80.6)	88.5 (76.2–100.7)	9	23	3	7	42
cN0 patients												
Groin parameters												
Visual assessment			62.5 (43.1–81.9)	71.1 (63.9–78.4)	69.9 (63.1–76.8)	25.9 (14.6–37.1)	92.2 (87.3–97.1)	15	106	9	43	173
Overall assessment			54.2 (34.2–74.1)	83.9 (78.0–89.8)	79.8 (73.8–85.8)	35.1 (19.8–50.5)	91.9 (87.3–96.5)	13	125	11	24	173
LN-SUV_max_	0.62 (0.46–0.78)	1.80, 0.34	45.8 (25.9–65.8)	87.9 (82.7–93.2)	82.1 (76.4–87.8)	37.9 (20.3–55.6)	91.0 (86.3–95.7)	11	131	13	18	173

*ROC*, receiver operating characteristic; *CI*, confidence interval; *PPV*, positive predictive value; *NPV*, negative predictive value; *TP*, true positive; *TN*, true negative; *FN*, false negative; *FP*, false positive; *LN*, lymph node

*According to ref. [28]

## Discussion

This retrospective study aimed to evaluate the diagnostic performance of preoperative [^18^F]FDG-PET/CT for LN staging in vulvar cancer patients. We selected diagnostic sensitivity and NPV as the favourite drivers for data analysis, considering how heavily a false negative result does worsen the prognosis, with the ultimate goal of removing as many as possible metastatic LNs, even at the cost of overtreatment. Since 2013, in our institution, we started to design clinical pathways entirely dedicated to women with vulvar cancer, focusing on a careful preoperative work-up with a very accurate assessment of the LN status, including PET/CT combined to standard imaging. In the current study, we included patients with vulvar cancer of any stage with the aim of exploring the absolute value of PET/CT in predicting the LN status. A subset analysis of cN0 patients was also included, given the dire consequences of under-treatment in these patients. Actually, the risk of missed LN metastases is less relevant in clinically/ultrasonography positive patients, who usually undergo bilateral dissection regardless PET/CT results.

We chose to apply and compare three different interpretative criteria of [^18^F]FDG-PET/CT images. All these methods, which reflect the common approach in routine clinical practice, showed good values of sensitivity and NPV at the groin level with no significant difference. Similar results were observed in the pelvis-based analysis. Previous PET or PET/CT studies showed variable results in terms of diagnostic performance, with sensitivity ranging from 50 to 100% and NPV from 57 to 100% for detecting metastatic LNs in vulvar cancer [12–20]. All these studies referred to small series (8–47 patients included) and mostly evaluated groin LNs. To our knowledge, the current study is the one with the highest number of patients and LN sites evaluated by PET/CT, having histopathology as reference standard. Some of the previous studies were prospective, analysing a selected subset of patients with different prevalence of metastatic LNs, which heavily influenced the predictive values of diagnostic results. In the study of Garganese et al., only patients with cN0 and invasive vulvar cancer who were candidates for radical inguinal surgery were investigated by [^18^F]FDG-PET/CT. The reported NPV was 93%, with a prevalence of metastatic groins of 12% [17]. We found similar NPV values at *visual assessment* both in the overall study population showing 30.4% prevalence of metastatic groins, and in the subgroups of cN0 patients, showing 13.9% prevalence of metastatic groins. From a clinical point of view, a high NPV predicts with great confidence the absence of LN metastases, thus suggesting that preoperative PET/CT is a valid support in better selecting patients suitable for minimally invasive inguinal surgery. We are aware that SNB is a useful minimally invasive surgical approach to explore LN status in vulvar cancer. However, SNB is recommended only in selected patients with cN0, according to strict criteria [4, 31]. In our series, among 96 cN0 patients, only 40 of them (41.7%) were candidate to SNB. As previously investigated by Garganese et al., a careful preoperative assessment of LN status by PET/CT combined with SNB could help to safely predict uninvolved inguinofemoral LNs, potentially extending the indication of minimally invasive LN surgery to a wider subgroup of patients, otherwise candidates for unnecessary diagnostic lymphadenectomy [17].

False negative LNs at [^18^F]FDG-PET/CT are usually associated with small metastatic foci in normal size nodes or in metastatic nodes with extensive necrosis. In our series, the median diameter of metastatic LNs with no [^18^F]FDG uptake was 5 mm. Therefore, a certain number of metastatic foci were under the limit of spatial resolution of PET/CT scanners. Also, the use of low-dose CT is suboptimal in detecting small metastatic or necrotic LNs. The addition of contrast-enhanced CT might help to evaluate other characteristics such as necrosis, non-homogenous enhancement and irregular margins, which are useful to distinguish benign versus malignant LNs.

When analysing the node size, there was a statistical difference in short axis size between metastatic and non-metastatic nodes both for normal and abnormal LNs at *visual assessment* as well as for clearly normal/inflammatory LNs (non-metastatic with high probability) and suspicious/clearly abnormal LNs (metastatic with high probability) at *overall assessment*. Therefore, the node size could be a variable in differentiating benign from metastatic nodes. As expected, the combination of PET and CT findings provided a significantly higher specificity (78.2% of *overall assessment* versus 65.5% of *visual assessment* at the groin level), but overall, did not contribute to accurately discriminate metastatic from non-metastatic LNs, due to a drop in sensitivity, although not significant. Therefore, given that our intention was to reduce the risk of missed LN metastases, CT criteria for positivity should not be used on top of visual interpretation of the PET images, as they reduce the number of true positive results.

In a smaller series of patients, we previously showed that for groin assessment, the use of delayed imaging (at 3 h from tracer injection) did not improve specificity and PPV with respect to standard imaging, highlighting that specificity is suboptimal, whatever method of analysis is used [19]. It is well known that increased [^18^F]FDG uptake is not specific for metastases, as inflammatory cells take up [^18^F]FDG. This can occur in inguinal reactive LNs after previous vulvar biopsy [32]. In our series, among 72 groins with negative LNs at histology and abnormal PET/CT at visual assessment, only 16 (22%) had been submitted to vulvar/LN biopsy in the 30 days prior to PET/CT with a median time of 15 days, thus suggesting that causes other than previous biopsy may be involved in false positive results at the LN level.

The ultimate goal of semi-quantitative analysis was to find a cut-off value of SUV_max_ able to discriminate metastatic from non-metastatic LNs. We found a higher cut-off value of SUV_max_ for pelvic LNs than for groin LNs (2.03 and 1.89, respectively). Even though one would expect a lower value for LNs located at a higher echelon, our finding is not surprising, given that both cut-off values refer to all the sites with positive LNs at histopathology. Actually, in our series, there were only 12 pelvic sites with pathologically positive LNs with a median SUV_max_ of 3.7; in the same patients, the median SUV_max_ at the groin level was 6.3, thus indicating an aggressive disease, spreading from the inguinofemoral to the pelvic LNs. When considering all the groins with positive LNs at histopathology, the median SUV_max_ was 3.5 and this justifies the cut-off value we have found, lower than that for pelvic LNs. Previous studies assessing the performance of [^18^F]FDG-PET/CT mainly used qualitative assessment [12–14, 17]. LN-SUV_max_ values were reported only in few manuscripts [15, 18–20]. In the current study, semi-quantitative analysis showed to be not superior to qualitative analysis, confirming our previous results in a small series of patients [19]. A recent study suggests that quantitative imaging metrics including SUV parameters, metabolic tumour volume and total lesion glycolysis may improve the diagnostic performance of PET/CT in identifying LN groin metastases from pelvic malignancies, including vulvar cancer [33].

The major limitation of our study is its retrospective nature and the consequent possible selection bias. Moreover, we did not take into account intra-patient correlation in the case of multiple lesions in a given patient, thus creating a clustered data structure. In any case, the complex regression models needed in this case are usually applied when a higher number of observations are considered [34]. Major strengths are the high number of patients, all with untreated vulvar cancer, and the high number of LN sites evaluated by PET/CT with a standardized approach. Another strength is the systematic evaluation of pelvic LNs, an issue that is not codified in the clinical practice. Recently, in a large series of vulvar cancer patients with pelvic nodal involvement, Shinde and co-workers showed that definitive locoregional therapy was associated with prolonged overall survival [35]. In this context, it is relevant to investigate pelvic LNs by imaging and/or histologic confirmation in order to tailor the treatment plan. Finally, at our institution, clinical and imaging information is routinely discussed during the Vul.Can MDT, including skilled physicians of the various specialties; this ensures a high-quality personalized approach.

## Conclusion

In a large series of vulvar cancer patients, preoperative [^18^F]FDG-PET/CT showed good sensitivity and NPV in discriminating metastatic from non-metastatic LNs, with values that are probably more reliable than those reported in the previous literature obtained in small and heterogeneous patient cohorts. In routine clinical practice, the use of qualitative analysis, in particular the simple *visual assessment* of PET data, is good enough making unnecessary the use of commonly used semi-quantitative parameters such as SUV_max_. In any case, a large prospective multi-centre study is recommended to confirm our data and assess the clinical value of [^18^F]FDG-PET/CT for LN staging, in order to personalise the surgical plan.

## Supplementary information

ESM 1 (DOCX 104 kb)
