# Supplementary material for: Diagnostic performance of preoperative [18F]FDG-PET/CT for lymph node staging in vulvar cancer: a large single-centre study
Source: Eur J Nucl Med Mol Imaging. 2021 Feb 23;48(10):3303–14. doi: 10.1007/s00259-021-05257-8 (PMC8426310; doi:10.1007/s00259-021-05257-8)
Supplement: Supplementary file 1 — (DOCX 104 kb) [file 259_2021_5257_MOESM1_ESM.docx]

**Article title** Diagnostic performance of preoperative [^18^F]FDG-PET/CT for lymph node staging in vulvar cancer: a large single-centre study

**Journal name** European Journal of Nuclear Medicine and Molecular Imaging

**Author names** Vittoria Rufini^*1,2^ MD and Giorgia Garganese^*3,4^ MD, Francesco P. Ieria^1^ MD, Tina Pasciuto^3^ EngD, PhD, Simona M. Fragomeni^3^ MD, Benedetta Gui^5^ MD, Anita Florit^1^ MD, Frediano Inzani^6^ MD, Gian Franco Zannoni^6,7^ MD, Giovanni Scambia^3,8^ MD, Alessandro Giordano^1,2^ MD, Angela Collarino^2^ MD, PhD.

^1^ Section of Nuclear Medicine, University Department of Radiological Sciences and Haematology, Università Cattolica del Sacro Cuore, Rome, Italy

^2^ Unit of Nuclear Medicine, Fondazione Policlinico Universitario A. Gemelli IRCCS, Rome, Italy

^3^ Department of Woman and Child Health and Public Health, Vul.Can MDT, Fondazione Policlinico Universitario A. Gemelli IRCCS, Rome, Italy

^4^ Gynecology and Breast Care Center, Mater Olbia Hospital, Olbia, Italy

^5^ Unit of Radiology, Fondazione Policlinico Universitario A. Gemelli IRCCS, Rome, Italy

^6^ Unit of Gynecopathology, Department of Woman and Child Health and Public Health, Fondazione Policlinico Universitario A. Gemelli IRCCS, Rome, Italy

^7^ Section of Pathology, Department of Woman and Child Health and Public Health, Università Cattolica del Sacro Cuore,

Rome, Italy

^8^ Section of Obstetrics and Gynecology, University Department of Life Sciences and Public Health, Università Cattolica del Sacro Cuore, Rome, Italy

*Contributed equally to this work.

Corresponding Author:

Prof. Vittoria Rufini, UOC Medicina Nucleare, Fondazione Policlinico Universitario A. Gemelli IRCCS, Largo A. Gemelli, 8 - 00168 Roma, Italia

Phone: +39.06.30154978; fax: +39.06.3058185; E-mail: [Vittoria.Rufini@unicatt.it](mailto:Vittoria.Rufini@unicatt.it)

ORCID: 0000-0002-1045-0633

| **Online Resource 1**: Diagnostic characteristics of 296 groin evaluated by PET/CT according to histopathology | | | | |
| --- | --- | --- | --- | --- |
| Characteristic | Whole study population | Positive groin lymph nodes at histology | Negative groin lymph nodes at histology | p value |
| **All cases** | n=296 | n=90 | n=206 |  |
| *Visual assessment* |  |  |  | **<0.0001** |
| 0 | 148 (50.0) | 13 (14.4) | 135 (65.5) |  |
| 1 | 63 (21.3) | 17 (18.9) | 46 (22.3) |  |
| 2 | 85 (28.7) | 60 (66.7) | 25 (12.1) |  |
| *Overall assessment* |  |  |  | **<0.0001** |
| Clearly normal | 148 (50.0) | 13 (14.4) | 135 (65.5) |  |
| Inflammatory | 32 (10.8) | 6 (6.7) | 26 (12.6) |  |
| Suspicious | 47 (15.9) | 15 (16.7) | 32 (15.5) |  |
| Clearly abnormal | 69 (23.3) | 56 (62.2) | 13 (6.3) |  |
| *SUV_max_* |  |  |  |  |
| Background | 1 (0.2) | 1 (0.2) | 1 (0.2) | 0.935 § |
| Lymph node * | 2.6 (0.9-28.4) | 4.1 (0.9-28.4) | 1.8 (0.9-8.1) | **<0.0001** |
| Lymph node † | 1.3 (0.5-28.4) | 3.5 (0.6-28.4) | 1.1 (0.5-8.1) | **<0.0001** |
| Results are presented as n (%) or mean (SD) or median (min-max) as appropriate. P values were calculated using Chi Square or Mann-Whitney test as appropriate, except where indicated. Bold font highlights statistically significant differences. § Evaluated with t-Student test (normally distributed characteristic). * Evaluated only in groins with score 1 and 2 at visual analysis. † Evaluated in all groins considering the lymph node SUV_max_ in groins with score 1 and 2, and the background SUV_max_ in groins with score 0 at visual analysis. | | | | |

| **Online Resource 2**: Diagnostic characteristics of 42 pelvic sites evaluated by PET/CT according to histopathology | | | | |
| --- | --- | --- | --- | --- |
| Characteristic | Whole study population | Positive pelvic lymph nodes at histology | Negative pelvic lymph nodes at histology | p value |
| **All cases** | n=42 | n=12 | n=30 |  |
| *Visual assessment* |  |  |  | **0.004** |
| 0 | 23 (54.8) | 3 (25.0) | 20 (66.7) |  |
| 1 | 9 (21.4) | 2 (16.7) | 7 (23.3) |  |
| 2 | 10 (23.8) | 7 (58.3) | 3 (10.0) |  |
| *Overall assessment* |  |  |  | **0.001** |
| Clearly normal | 23 (54.8) | 3 (25.0) | 20 (66.7) |  |
| Inflammatory | 0 (0) | 0 (0) | 0 (0) |  |
| Suspicious | 8 (19.0) | 1 (8.3) | 7 (23.3) |  |
| Clearly abnormal | 11 (26.2) | 8 (66.7) | 3 (10.0) |  |
| *SUV_max_* |  |  |  |  |
| Background | 1.0 (0.7-1.5) | 1.1 (0.9-1.5) | 1.0 (0.7-1.4) | 0.309 |
| Lymph node * | 3.1 (1.8-15.5) | 3.7 (2.2-15.5) | 2.5 (1.8-6.7) | **0.034** |
| Lymph node † | 1.4 (0.7-15.5) | 3.7 (1.1-15.5) | 1.1 (0.7-6.7) | **0.0006** |
| Results are presented as n (%) or median (min-max) as appropriate. P values were calculated using Chi-Square or Mann-Whitney test as appropriate. § Evaluated with t-Student test (normally distributed characteristic). Bold font highlights statistically significant differences. * Evaluated only in pelvic sites with score 1 and 2 at visual analysis. † Evaluated in all pelvic sites considering the lymph node Suv_max_ in sites with score 1 and 2, and the background SUV_max_ in sites with score 0 at visual analysis. | | | | |

| **Online Resource 3:** Results of z test of proportion according to diagnostic performances parameters | | | | | |
| --- | --- | --- | --- | --- | --- |
| Parameters | p value for sensitivity | p value for specificity | p value for accuracy | p value for PPV | p value for NPV |
| **Groin** |  |  |  |  |  |
| LN SUV_max_ vs. *visual assessment* | 0.049 | **<0 .00001** | **0.0101** | 0.029 | 0.435 |
| LN SUV_max_ vs. *overall assessment* | 0.596 | 0.271 | 0.549 | 0.516 | 0.779 |
| *Overall assessment* vs. *visual assessment* | 0.242 | **0.003** | 0 .049 | 0.121 | 0.603 |
| **Pelvis** |  |  |  |  |  |
| LN SUV_max_ vs. *visual assessment* | 1.000 | 0.195 | 0.273 | 0.600 | 0.873 |
| LN SUV_max_ vs. *overall assessment* | 1.000 | 0.195 | 0.273 | 0.600 | 0.873 |
| *Visual assessment* vs. *overall assessment* | 1.000 | 1.000 | 1.000 | 1.000 | 1.000 |
| Bold font highlights statistically significant differences. Bonferroni multiple testing correction was applied with a significant level set at 0.017. | | | | | |
